# A Real-Time Marker-Based Visual Sensor Based on a FPGA and a Soft Core Processor

**DOI:** 10.3390/s16122139

**Published:** 2016-12-15

**Authors:** Hilal Tayara, Woonchul Ham, Kil To Chong

**Affiliations:** 1 Department of Electronics and Information Engineering, Chonbuk National University, Jeonju 54896, Korea; hilaltayara@jbnu.ac.kr (H.T.); wcham@jbnu.ac.kr (W.H.); 2 Advanced Electronics and Information Research Center, Chonbuk National University, Jeonju 54896, Korea

**Keywords:** coplanar PosIt, FAST corner detection, FPGA, image segmentation, visual sensors

## Abstract

This paper introduces a real-time marker-based visual sensor architecture for mobile robot localization and navigation. A hardware acceleration architecture for post video processing system was implemented on a field-programmable gate array (FPGA). The pose calculation algorithm was implemented in a System on Chip (SoC) with an Altera Nios II soft-core processor. For every frame, single pass image segmentation and Feature Accelerated Segment Test (FAST) corner detection were used for extracting the predefined markers with known geometries in FPGA. Coplanar PosIT algorithm was implemented on the Nios II soft-core processor supplied with floating point hardware for accelerating floating point operations. Trigonometric functions have been approximated using Taylor series and cubic approximation using Lagrange polynomials. Inverse square root method has been implemented for approximating square root computations. Real time results have been achieved and pixel streams have been processed on the fly without any need to buffer the input frame for further implementation.

## 1. Introduction

Computer vision (CV) has many possible utilizations and is one of the fastest growing research areas these days. Passive optical sensors have been used in the automotive industry for tasks such as management and off-board traffic observation [1]. The CV technique is often used in driver assistance and traffic sign detection in onboard systems [2]. Also, the augmented reality (AR) resulting from CV functions, improves the real environment by using virtual elements and allows diversified functions such as guided order picking or upkeep efforts [3,4].

Various light conditions, different views of an object, surface reflections and noise from image sensors are the challenging questions with which one has to deal when it comes to pose estimation and optical object detection. The solution to these problems to some extent can be achieved thanks to the use of SIFT or SURP algorithms as they compute the point features, which are invariant towards scaling and rotation [5,6]. However, these algorithms demand powerful hardware and consume high battery power because of their high computational complexity, making their use in automotive applications and in the field of mobile devices generally challenging.

The Nios II microprocessor has been widely used in many applications such as image processing, control, and mathematic acceleration. Low-cost sensors have been discussed in [7]. They have accelerated block matching motion estimation techniques using the Altera C2H. Their processor can process 50 × 50 at 29.5 fps. Custom instructions in the Nios II were used for accelerating block matching algorithms [8] where a 75% improvement was achieved by utilizing custom instructions. In [9], customized Nios II multi-cycle instructions have been used to accelerate block matching techniques. A Nios II microprocessor was also used for image encryption and decryption. The AES algorithm was implemented using a Nios II soft core processor [10]. The proposed implementation provides high security while retaining image quality. A MPPT controller for a hybrid wind/ photovoltaic system was developed using a Nios II microprocessor [11]. In addition, a Nios II microprocessor was used to accelerate a quadratic sieve (QS) algorithm to factor RSA numbers [12].

The task of camera pose estimation has been addressed by several algorithms. These algorithms can be categorized into two groups: iterative and analytical [13,14,15]. Analytical algorithms utilize closed solutions with some assumptions in order to simplify this problem [16,17]. Iterative methods compare the projection with actual measured correspondences. Iterative methods are more used due to their accurate results and minimal number of iterations [13,14,15,18,19,20]. In this paper, the Nios II processor is used for estimating the pose based on [19].

This paper describes a real time system to recognize a predefined marker with known geometries and calculate the pose of the detected markers in the frame. This system is divided into two main subsystems. The first one is carried out on FPGA and used for extracting the four vertices of the recognized markers which are required for pose estimation. The markers are recognized in the frame by using single pass image segmentation and a FAST algorithm. The second subsystem is a Nios II soft core processor based on RISC architecture. Using this processor, the Coplanar PosIT algorithm can be implemented. In order to achieve the fast use of floating point operations, additional floating point hardware was added to this processor. Moreover, the time required for running Coplanar PosIt has been reduced by approximating trigonometric functions using Taylor series and Lagrange polynomials. The entire system has been implemented on a Cyclone IVE EP4CE115 chip from Altera (San Jose, CA, USA) which operates at 50 MHZ. Quartus and Qsys were used for system building.

This paper is divided into the following sections: Section 2 explores the related works. Section 3 explains the top level architecture. Section 4 presents the detailed architecture of the proposed system. Simulation results are discussed in Section 5. Section 6 gives the conclusions.

## 2. Related Works

Natural features of images, including global or local features, can been used in object recognition because of the increasing processing power of traditional PC hardware [21]. Feature recognition techniques have been used in developing many applications such as autonomous systems and automotive systems [22]. Harris Corner Detection, SIFT, and SURF are well-known algorithms for finding and describing point features [5,23]. SIFT and SURF extract local features which outperform global features [6,24]. However, the speed of SURF is faster than SIFT. The main drawback of the aforementioned algorithms is the need for high computational power which requires powerful PC-like hardware [21], making the availability of high-speed platforms with such powerful PC-like hardware a necessity to implement these algorithms. A modern GPU hardware and programmable logic (FPGAs) have been used to meet the computational requirements of these algorithms [25]. Histogram of Oriented Gradient (HOG) for object detection has been implemented using reduced bit width fixed point presentation in order to reduce required area resources. This implementation was able to process 68.2 fps for 640 × 480 image resolution on single FPGA [26]. Another reduced fixed point bit width for human action recognition has been introduced in [27] where hardware requirements have been reduced due to using a classifier with 8 bit-fixed-point. Bio-inspired optical flow has been implemented in reconfigurable hardware using a customizable architecture [28,29]. FPGA was used in order to enhance the performance of a localization algorithm. FAST corner detection has been accelerated using a FPGA-based grid approach in [30]. Mirage pose estimation has been used for robot localization and trajectory tracking. Pose was estimated analytically without iterative solutions [31]. A POSIT algorithm for non-coplanar feature points has been used for UAV localization in [32].

A SURF-based object recognition and pose estimation embedded system was introduced in [33]. This system is able to process images at five frames per second and recognize nine different objects. However, this system is not suitable for real-time systems due to the latency produced by the SURF algorithm implementation. On-board vision sensors have been designed and implemented using a linked list algorithm in [34]. In this work, memory consumption had been minimized and the proposed algorithm is able to detect blobs in real-time.

## 3. Architecture of the Proposed System

**Figure 1:**
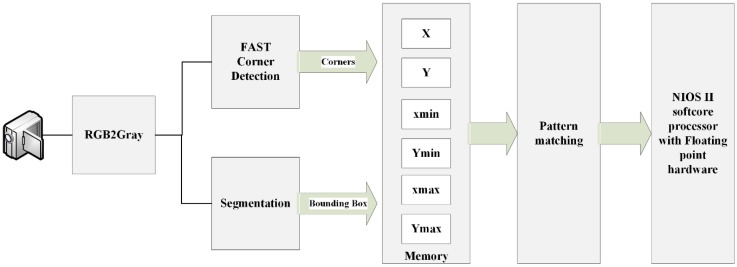
Top level architecture.

**Figure 2:**
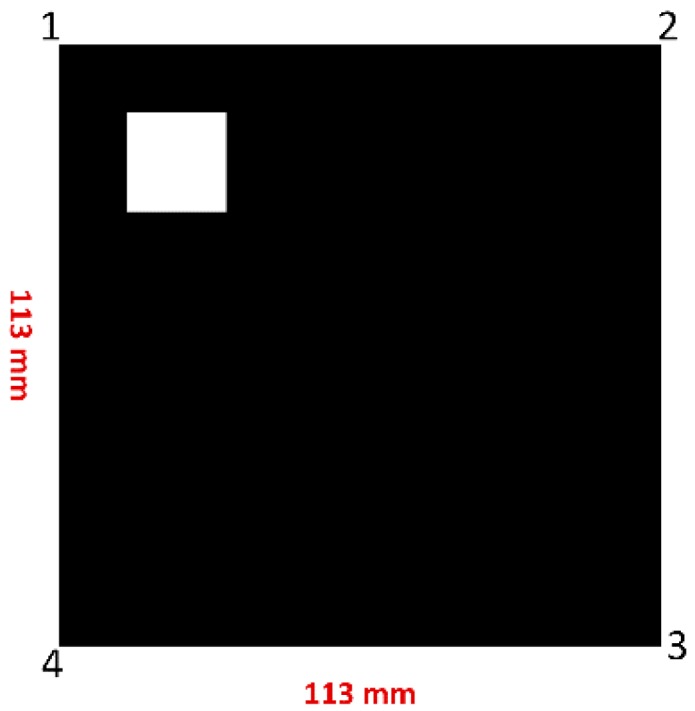
Marker properties.

The proposed system has five main stages as shown in Figure 1. It starts by converting the input pixel stream into grayscale which will be the input for the next stage. The grayscale level is represented by 8-bits. In the second stage, image segmentation and FAST corner detection are performed on parallel for extracting foreground objects and objects corners, respectively. The results of the second stage will be bounding box coordinates for every detected object and its corners. Bounding box coordinates and corners coordinates will be stored in the third stage which is implemented by M9K block memories. Since the main target of the proposed system is fast implementation, a simple marker has been chosen as shown in Figure 2. The marker geometries are predefined, with each side measuring 113 mm. This marker will reduce the matching time and lead to high-speed implementation. The output of pattern matching stage is four vertices of the detected markers which will be sent to the pose estimation stage. These vertices should be ordered as shown in Figure 2. The order of marker’s vertices is important for pose estimation procedure. In the final stage, NIOS II soft-core processor will be used for executing the coplanar PosIt algorithm to find out the pose of the detected markers. In the following section, a detailed explanation about the components of the proposed system is given.

## 4. Detailed Architecture of the Proposed System

### 4.1. Grayscale

Grayscale pixels are needed to perform image segmentation and corner detection. In order to implement this subsystem in hardware efficiently, red and blue components were divided by four, through shifting two digits to the right. Whereas green component was divided by two, by shifting one digit to the right. The summation of the shifted components gives grayscale level as shown in Equation (1) where “>>” means logical right-shift.

Grayscale = R>>2 + G>>1 + B>>2(1)

### 4.2. Segmentation

**Figure 3:**
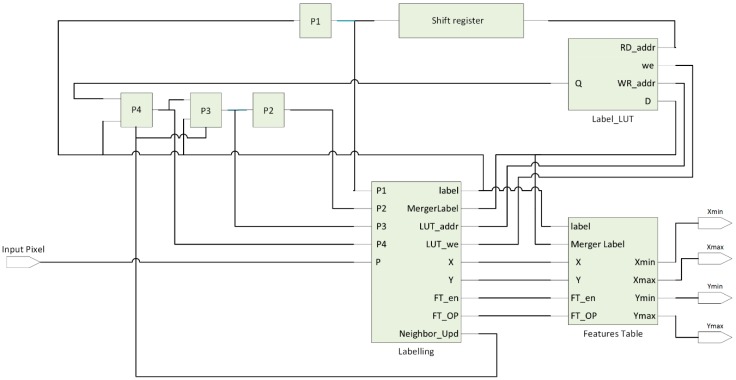
Single pass image segmentation architecture.

One of the most central image analysis tools is Blob analysis; it is used in marker-based tracking applications. The major idea of using blob analysis is extracting the foreground regions from the frame and calculating their geometric features. Talking about the classical algorithms, two or three successive raster scans through the image are required [35,36]. A temporary label is designated to the current foreground pixel based on the labels of the neighbors that have already been inspected in the first scan. Different labels can be used with the same blob [37]. In the second pass, the replacement of the temporary label is required by its equivalent. In the final pass, the features of each blob are computed based on the blob information such as area, bounding box, and orientation. It is clearly determined that the classical algorithms are not convenient for hardware implementation due to the following reasons, conversion of these algorithms to parallel is severe because of their sequential nature. Secondly, a complete image frame should be buffered before the next pass starts. To achieve this, we need high memory bandwidth and long processing delays, which is not acceptable in applications where the system latency is critical. In the proposed system, it is required that pixels should be processed on the fly to ensure minimum latency. As a result, single pass image segmentation architecture was adopted to fulfill this constraint. The single pass image segmentation architecture consists of four main blocks, as shown in Figure 3. It is based on the architecture suggested in [38]. This architecture has the following blocks.

#### 4.2.1. Neighborhood Block

This block is used for dealing with the neighborhood pixels. The neighbor pixels are stored in the registers P1, P2, P3 and P4. These registers are shifted at each clock cycle during the window scanning across the image. A row buffer is used for storing the last processed row of the image since the resulting labeled image is not stored. The labels of this row buffer need to be updated from the “Label_LUT” before being used in processing further input pixels of the image. Multiplexers will be added before P3 and P4. When there is a merger case the value of P3 should be updated by the new label value. For pixel P4, the next value of this pixel is being read out at the same time the “Label_LUT” is being updated which means that the value read from the “Label_LUT” is the label before the merger. Consequently, if the next value for P4 is not background pixel, the newly merged label should be written in P4.

#### 4.2.2. Label_LUT

Output of the shift register will be assigned to “RD_address” of the “Label_LUT” block to assure that the appropriate label will be used for any label stored in the shift register. Initialization of the “Label_LUT” before the processing is not required because at any time when there is a new label, a new entry will be added in the “Label_LUT” pointing to itself. Whenever a new merger case appears, this LUT will be updated by the labeling block.

#### 4.2.3. Labeling Block

Based on the neighboring pixels the labeling of the current pixels takes place making the use of following algorithm [36]:

- Assign zero when the input pixel is background.
- Assign new label when all neighbors are backgrounds.
- In case of only one label is used in the labeled neighbors, this label will be assigned to the current pixel.
- A merger condition occurs when there are two different labels used among the neighbors.

**Figure 4:**
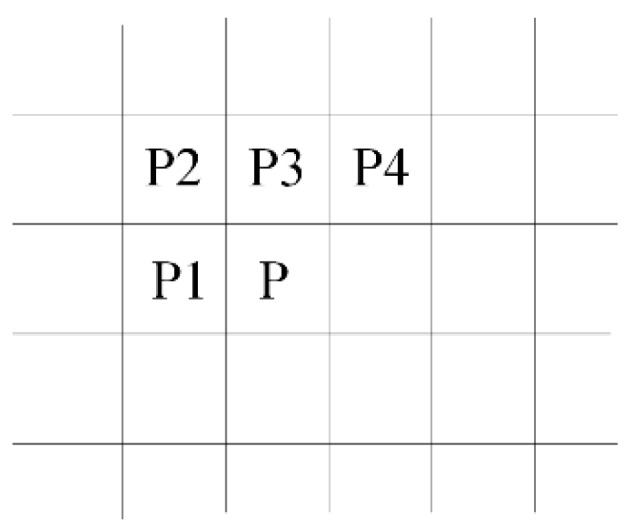
8-connectivity pixel mask.

By understanding that image segmentation algorithm processes foreground pixels, the overall execution time is reduced by filtering out the background pixels that require no further process. The fact that the neighboring pixels are not independent, the processing of foreground pixels can be optimized [39]. For example, if the pixel P3—which is the neighbor of all the pixels in the mask—is foreground pixel, all pixels in the neighborhood must share the same label of P3. This shows that the number of the neighbors to be inspected is decreased from four to one. By studying the 8-connectivity pixel mask which is shown in Figure 4, a total of thirty-two cases will appear. These cases can be classified into ten categories, as listed in Table 1.

These categories can be explained as follows:

- New Label: in this case, a new label is created and assigned to the input pixel P. A new entry will be created in “Label_LUT”. A new entry in “Features Table” for the newly created label will be added. The number of blobs will be incremented by one.
- Copy P1: in this case, the label of P1 will be assigned to P. The corresponding features values in “Features Table” at P1 address will be updated. “Label_LUT” will not be updated. A similar process will occur for Copy P2, Copy P3 and Copy P4.
- Compare P1 and P4: If P1 equals P4, P1 will be copied to the pixel P and features values at P1 address will be updated. “Label_LUT” will not be updated. If P1 < P4, a merger condition occurs. In this case, P1 will be copied to the pixel P, features values in the “Features Table” at P1 and P4 will be merged and stored at P1 address and “Label_LUT” will be updated by replacing the value at P4 with the value at P1. In addition, multiplexers will be set to read the newly updated label. When P4 < P1, a merger condition takes place. In this case, P4 will be copied to the pixel P, features values in the “Features Table” at P1 and P4 will be merged and stored at P4 and “Label_LUT” will be updated by replacing the value at P1 with the value of P4. Multiplexers will be set to read the newly updated label. The number of blobs will be decremented by one when there is a merger case. A similar process will occur for “Compare P2 and P4”.
- Leave Blob: Labeling block has a lookup table called “Last_x“ which is used for storing the x-coordinate of the pixel P. This “Last_x” LUT is used for deciding the “finish blob” case.
- Finish Blob: If the value of Last_x[P2] equals to the current x-coordinate of the input pixel, the current blob is finished, bounding box coordinates of the current region can be sent to next processing step and the label can be reused again.
- NOP: No operation.

#### 4.2.4. Features Table

The last component to be discussed in image segmentation block is the features table. It is updated for every processed pixel. In the proposed system, the bounding box is the only required feature for every blob. Four Dual Port Rams (DPR) are used for storing Xmin, Xmax, Ymin and Ymax for every extracted region. For updating the bounding box feature for every region in the image, two states have been considered in this architecture.

- State0: In this state, the feature values at the addresses “Label” and “Merger Label” will be read out. After that, according to the “opcode” from the labeling block the value that will be written back in DPR’s will be figured out. A two-bit op code is used for representing four possible cases as follows: op = 00, new blob is recognized, the value of x and y coordinates of this blob will be prepared to be stored in the DPR’s in the next clock cycle of the features table’s frequency. When op = 01, the bounding box information of the blob defined by “label” will be updated. When op = 10, a merger case occurred, the data read out from port A and port B will be compared and the minimum one will be selected as xmin/ymin and the maximum one will be selected as xmax/ymax. When op = 11, the current blob completes and the data read out from DPR’s will be sent to the next processing step.
- State1: the data prepared from the state0 will be written in the DPR’s. Algorithm 1 shows the pseudo code for updating xmin/ymin feature.

The merger table requires three clock cycles for performing one merge. However, since dual-port ram is used, these three steps are pipelined with a throughput with one merger per clock cycle.

**Algorithm 1.** Pseudo code for updating xmin/ymin feature.Always @(posedge clk) beginIf state==0 then  //Read feature values at label and merger_label  rd1 = portA[label];  rd2 = portB[merger_label];  case (op)   00: wd1 = x; // new block recognized   01: if x < rd1 then //update the current blob     wd1 = x;   10: wd1 = (rd1 < rd2)? rd1:rd2; // merger case occurred   11: send rd1 to next processing stage; // end of the current blob  endcase  state = 1else  portA[label] = wd1; // writing the prepared data in the state0 in the DPR.  State = 0;endend

### 4.3. FAST Corner Detection

**Figure 5:**
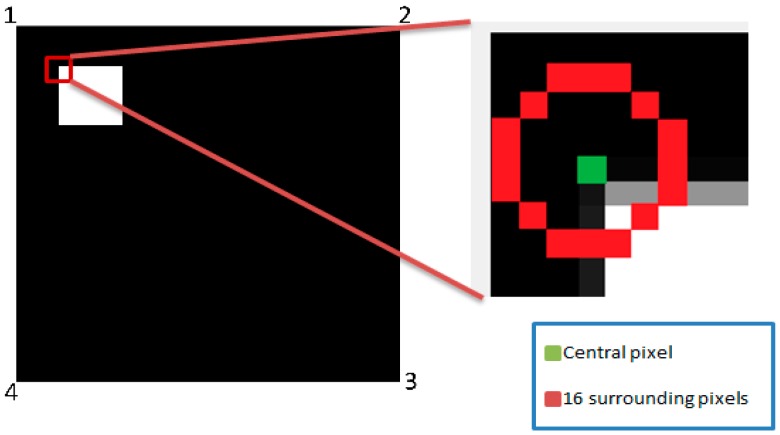
Illustration of the central pixel and 16 surrounding pixels.

In the proposed system, a Features from Accelerated Segment Test (FAST) algorithm was used since it provides high-speed corner detection and requires fewer hardware resources compared to other corner detection algorithms such as Harris corner detection. FAST was introduced by Rosten and Drummond [40,41]. FAST algorithm can be summarized in the following steps:

1. Select a pixel P with intensity Ip.
2. Set an appropriate threshold T.
3. Get a circle of sixteen pixels around the pixel under test as shown in Figure 5.
4. Decide that the central pixel P is a corner if there exists a set of n contiguous pixels in the surrounding circle which are all brighter than Ip + T, or darker than Ip − T where Ip is the intensity of the central pixel and T is the selected threshold.

**Figure 6:**
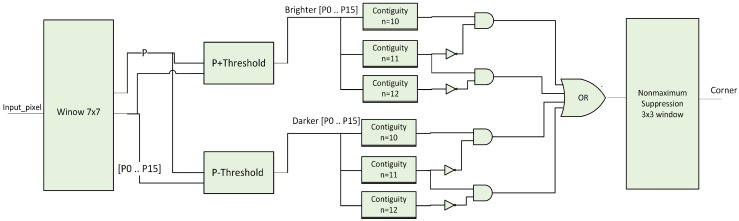
FAST architecture.

FAST corner detection consists of a two-stage pipeline. The first stage is thresholder block and the second one for n contiguity checking. This pipelined implementation is efficient for determining in each clock cycle if a pixel is a corner or not. The subsystem of FAST corner detection is given by Figure 6.

- Window 7 × 7: this block is used for getting the 16 surrounding pixels of the circle and the central pixel. In order to achieve this, six FIFO delay buffers were used, the width of every FIFO equals to the image width. In addition, 42 8-bit registers were used to store pixel intensity values in the investigated window. The 16 pixels and the central pixel will pass to the thresholder stage.
- Thresholder: this block decides if the pixels in the circle are brighter or darker than the central pixel. If the intensity of the pixel Pi-Pi is a pixel in the circle- is greater than the intensity of the central pixel P added to threshold then the pixel Pi is brighter than the pixel P. In this case, it will be assigned 1 in the output vector in the corresponding position of the pixel Pi. On the other hand, if the intensity of the pixel Pi is smaller than the intensity of the central pixel P subtracted by threshold then the pixel Pi is darker than the pixel P. It will be assigned 1 in the output vector in the same position of the pixel Pi. As a result, two 16-bit vectors will be the result of this stage. One is for the brighter pixels and the other one is for the darker pixels.
- Contiguity blocks: these blocks are used to study if there is n contiguous bright or dark pixels in the circle. In this system, three blocks were used for studying the contiguity n = 10, n = 11 and n = 12. The architecture for each block contains 16 n-input logical AND gates. The output of these contiguity blocks will be set to 1 if there is a ‘10’ or ‘11’ contiguous bright or dark pixels in the circle.
- OR gate: this gate is used for deciding whether the pixel is a corner or not from brighter or darker contiguities blocks.
- Non-maximum suppression: the 3 × 3 window was used with two FIFOs and six 1-bit registers. The architecture of this block is similar to the window 7 × 7 block.

### 4.4. Memory Blocks

In this block, six memory blocks were used to store the coordinates of detected corners from FAST block and the coordinates of bounding box from single pass image segmentation block. For x, y coordinates of the extracted corners, the memory depth is 1024 and the word width is 10 bits. For bounding box coordinates, four memory blocks were used for storing Xmin, Xmax, Ymin and Ymax. In this system, 256 objects could be extracted from the image and word width is 10 bits. M9K blocks where used to implement those memory blocks.

### 4.5. Pattern Matching

The main goal in this stage is to make matching process as simple as possible in order to achieve real time implementation. For every extracted region, the location of the corners will be used for deciding whether the region is the predefined marker or not. The general steps followed for extracting the vertices of the detected markers are:

- Divide the bounding box into four quarters by calculating the center coordinates of the bounding box.
- Every quarter should have one corner, located only on the edge of the bounding box.
- It is assumed that all corners of the inner square are located in one quarter or in two adjacent quarters.
- Based on the location of the inner square, the first vertex of the marker will be decided.
- The remaining vertices are a clockwise direction to the first one.

**Figure 7:**
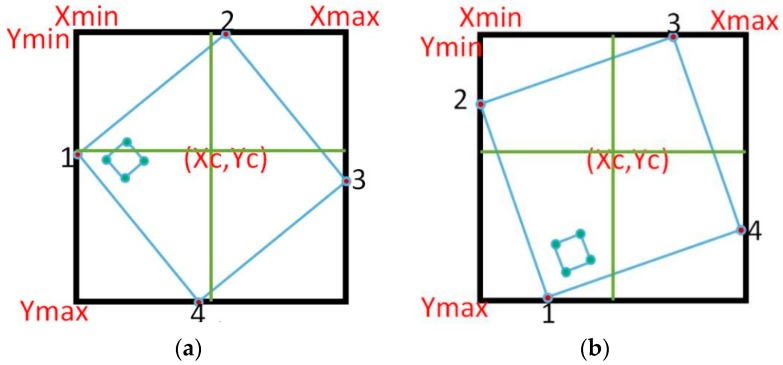
Illustration of marker detection (**a**) inner corners are located in two adjacent quarters; (**b**) inner corners are located in one quarter.

Figure 7 shows illustration of marker detection.

### 4.6. Pose Estimation

The Coplanar PosIt algorithm was used in order to estimate the pose of the detected markers. This algorithm was developed by Daniel DeMenthon [19]. It works by comparing some coplanar point coordinates with predefined object points and statistically finding the best rotation and translation that fit the object projection in these points.

#### 4.6.1. Coplanar PosIt Algorithm

**Figure 8:**
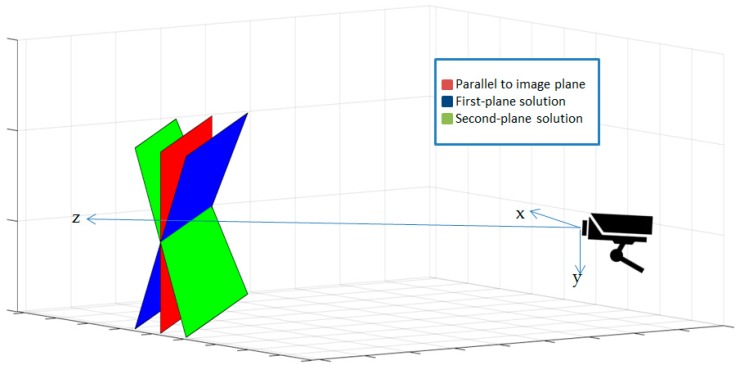
Shows how object plane 1 and object plane 2 looks the same seen from the camera, and the planar version of PosIt returns both these solutions.

**Figure 9:**
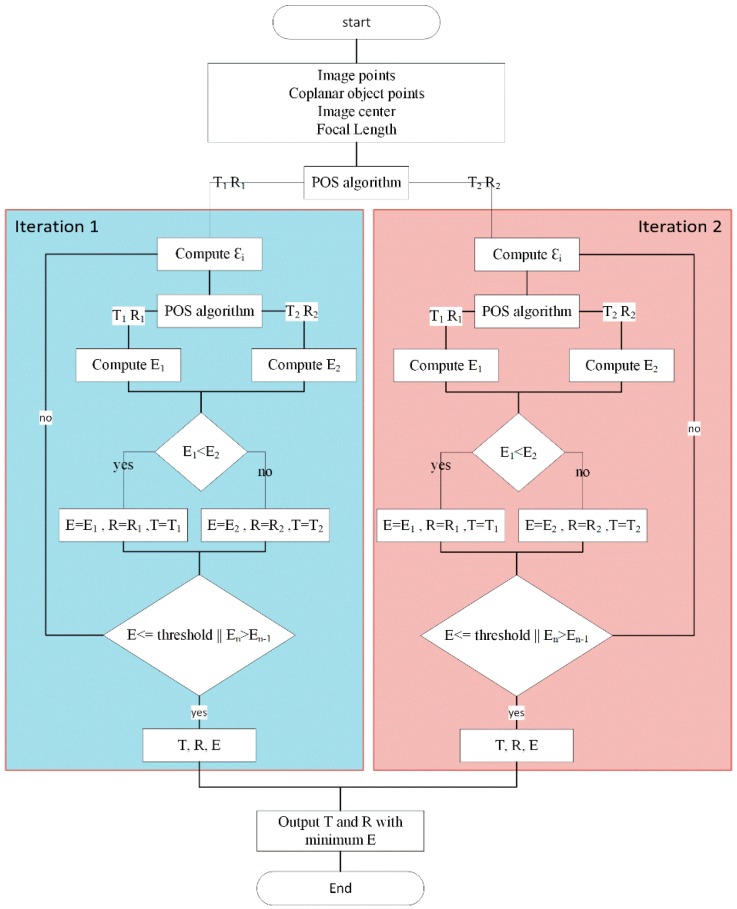
Coplanar POSIT flowchart.

The Coplanar PosIt algorithm starts by finding the pose from orthography and scaling, which approximates the perspective projection with a scaled orthographic projection and finds the rotation matrix and translation vector from a linear set of equations. After that, PosIt iteratively uses a scale factor for each point to enhance the found orthographic projection and then uses POS algorithm on the new points instead of the original ones until the threshold is met. However, dealing with planes will give more poses for same orthographic projection as show in Figure 8. Two branches will be created after the first iteration of the algorithm. Every branch will be assigned with one solution produced from the first iteration. After that, the best pose will be kept for each branch. The best solution will be decided based on measuring the average Euclidean distance (E) between the actual image points and corresponding predicted image points for the found pose divided by the image diagonal. The iteration stops if E is smaller than a predefined threshold or a certain number of iterations were achieved. Figure 9 shows the flow chart of the implemented algorithm on Nios II soft core processor.

#### 4.6.2. Soft-Core Processor

**Figure 10:**
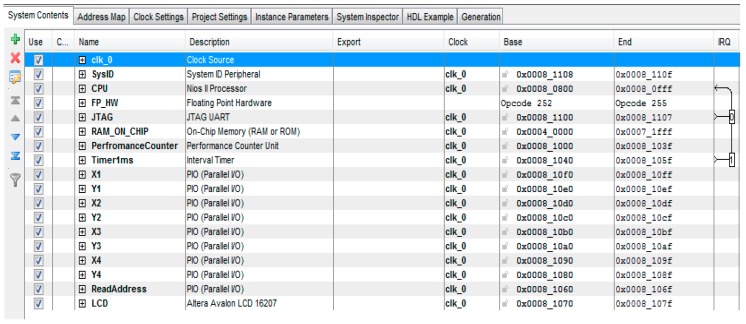
Soft-core processor components and architecture.

**Figure 11:**
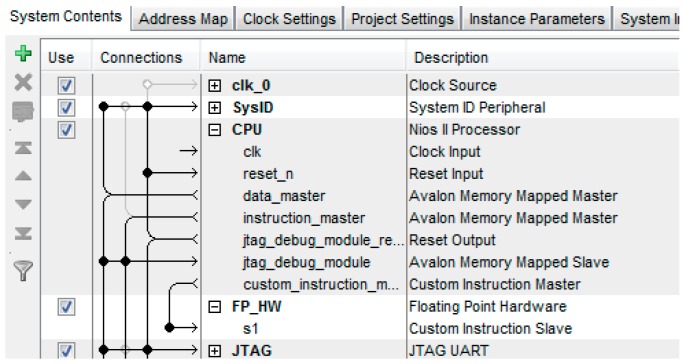
Connecting floating point hardware with the Nios II CPU.

The Coplanar PosIt algorithm was implemented by using z Nios II soft-core processor which was built using Qsys. Qsys is a tool provided by Altera which optimizes time and effort in the FPGA design process. A complete and complex system could be created by using the peripherals available in the Qsys libraries [42]. Qsys provides faster development, faster timing closure and faster verification. The proposed soft-core processor system is shown in Figure 10. The clock frequency of the proposed system is 50 MHz. A NIOS II/f processor with hardware multipliers and hardware dividers is used. The NIOS II/f processor is a high-performance 32-bit processor based on a Reduced Instruction Set Computer (RISC) architecture. The fastest version of this processor, which allows utilization of entire instruction set available, is used. In order to accelerate the arithmetic functions executed on float variables to find the pose, NiosII Floating Point Custom Instructions (FPCI) is used. FPCI implements single-precision floating-point arithmetic operations. The existence of FPCI in the system forces the compiler to use the custom instructions for floating point operations. The ca ustom instruction master of NiosII CPU is connected with custom instruction slave in the Floating Point Hardware as shown in Figure 11. The size of on chip RAM is 200 KB. Eight parallel I/O (PIO) ports are used for reading the coordinates of the four vertices of the detected markers. The width of these ports is 10 bits and they act as input ports. One PIO is used as output port with a width equals to 8 bits in order to be used as read address for the vertices stored in the memory blocks. In addition, performance counter component is used for studying the performance of the system.

#### 4.6.3. Floating-Point versus Fixed-Point

Fixed-point representation is a way of implementing floating-point operations using ordinary integer representation. In other words, a fixed-point number is a scaled version of a floating-point number. It is defined by word length in bits, position of the binary point, and whether it is signed or unsigned. This position affects the range—the difference between the largest and smallest representable numbers—and precision -the smallest possible difference between any two numbers. The main advantage of using fixed-point is its simplicity since, we can use same hardware that does integer arithmetic in fix-point arithmetic. However, using fixed-point in implementing an algorithm is error-prone due to following reasons: (i) deciding where the position of the fixed-point of the variables requires good understanding of the algorithm; (ii) Infinities are not represented by fixed-point; (iii) Overflow should be predicted and manually managed; (iv) Rounding toward-∞ for signed and unsigned arithmetic due to truncation since it is the cheapest rounding mode; (v) Multiplying two integers with the same size produces a result with twice the size of inputs.

On the other hand, floating-point numbers are accurate approximation of real numbers and easy to use. There are three components for representing floating-point numbers including, sign (S), exponent (E) and the fraction—mantissa—(M). IEEE standard 754 set up in 1985 a uniform standard for floating-point arithmetic. This standard has three precisions including single precision 32 bits (S = 1, E = 8 and M = 23), double precision 64 bits (S = 1, E = 11 and M = 52), and extended precision 80 bits (S = 1, E = 15 and M = 64) [43]. Nios II floating point custom instruction unit supports the IEEE standard 754 [42].

#### 4.6.4. Mathematical Functions Approximation

Pose estimation algorithms require calculating some trigonometric functions like sine, cosine, arc cosine and arctangent. These functions consume long execution times. In order to reduce this time, these functions have been approximated using Taylor series and cubic approximation using Lagrange polynomials. Sine, cosine and arctangent were approximated using Taylor series, however, the best approximation for arc cosine was a Lagrange polynomial. Sine function has been approximated as shown in Equation (2). Cosine function has been approximated using Equation (3), while arc cosine and arctangent have been approximated using Equations (4) and (5), respectively. In addition to trigonometric functions, square roots are also required for estimating the pose of the detected markers. However, this function requires a long execution time too, hence, fast inverse square root was utilized. This method uses Newton’s method of approximation but it starts with a guess very close to the solution. Only one iteration is enough for Newton’s method to get a good enough solution [44]:

(2) $$\sin(x)\approx x-\frac{x^{3}}{3!}+\frac{x^{5}}{5!}-\frac{x^{7}}{7!}$$

(3) $$\cos(x)\approx 1-\frac{x^{2}}{2!}+\frac{x^{4}}{4!}-\frac{x^{6}}{6!}+\frac{x^{8}}{8!}$$

(4) $$a\cos(x)\approx (-0.6981317x^{2}-0.8726646)x+1.5707963$$

(5) $$a\tan(x)\approx \{\begin{array}{cc} x-\frac{x^{3}}{3}+\frac{x^{5}}{5}-\frac{x^{7}}{7} & -1<x<1\\ \frac{\pi }{2}-\frac{1}{x}+\frac{1}{3x^{3}}-\frac{1}{5x^{5}} & x>=1\\ -\frac{\pi }{2}-\frac{1}{x}+\frac{1}{3x^{3}}-\frac{1}{5x^{5}} & x<=-1 \end{array}$$

## 5. Experimental Results

**Figure 12:**
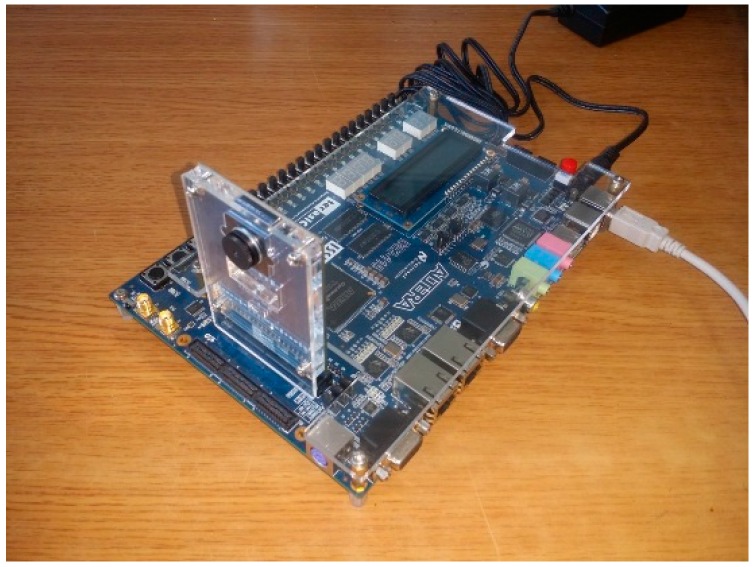
Experimental equipment.

System experiments and simulations where carried out using an Altera DE2-115 board powered by a Cyclon IVE FPGA chip. Figure 12 shows the experimental equipment used in this experiment.

**Figure 13:**
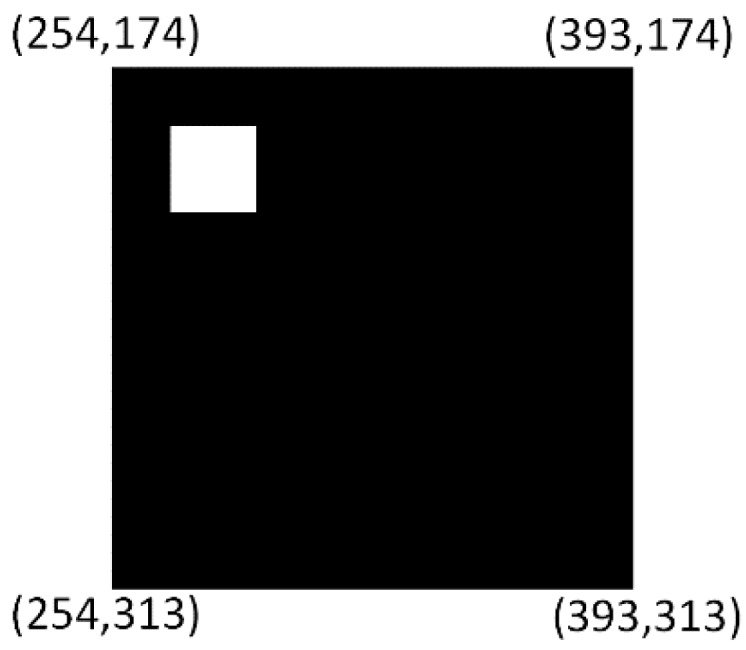
Illustration of input frame with one marker located in the center.

The simulation results for a frame containing one marker in the center of the frame will be introduced. The illustration of this frame is shown in Figure 13.

**Figure 14:**
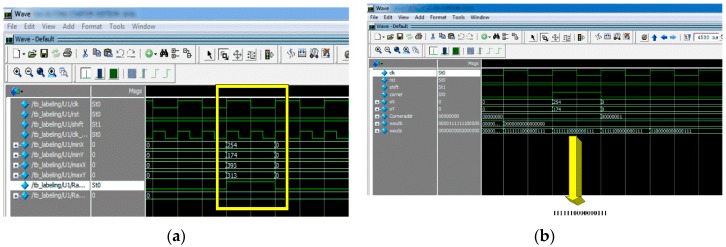
(**a**) Bounding box coordinates; (**b**) Corner Detection using FAST.

Figure 14a shows the bounding box coordinates of the detected marker. It can be seen from this figure that the coordinates of the bounding box [Xmin, Ymin, Xmax, Xmax] = [254, 174, 393, 313]. In addition, write enable is set to 1 in order to write these values in the corresponding memories.

In addition, detected corners using FAST algorithm are shown in Figure 14b. It can be seen that the ‘corner’ signal is set to one because there is a contiguity pattern equals to ‘1111111000000111’. In this case *n* = 10. The detected corner at (X = 254, Y = 174) will be written at the address specified by ‘Coneraddr’.

**Figure 15:**
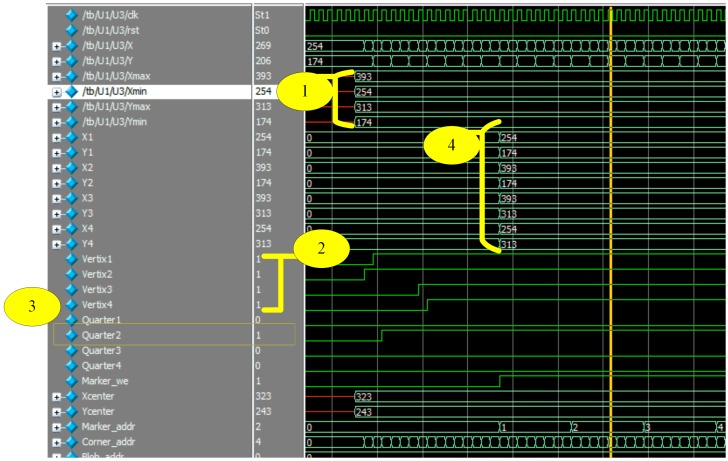
Matching process. . “1” illustrates reading out the coordinates of the bounding box. “2” illustrates the vertices found in the bounding box, “3” illustrates that the first vertex is located in quarter 2, and “4” illustrates the output of the ordered vertices.

The matching process is illustrated in Figure 15. This figure is marked from one to four. Step 1 represents reading coordinates of the bounding box. Step 2 shows how vertices signals became 1 during the reading of corner coordinates. Step 3 depicts that, the first vertex is located in the second quarter. Step 4 shows the output of the ordered vertices where [V1:(254,174), V2:(393,174), V3:(393,313), V4:(254,313)].

**Figure 16:**
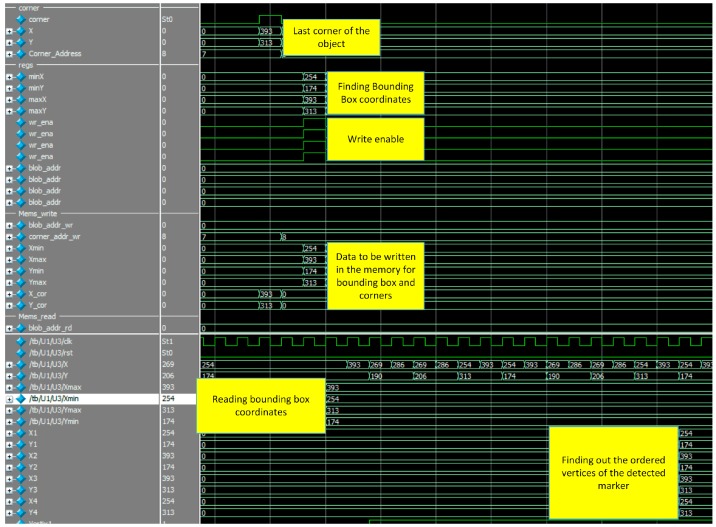
Whole system simulation results.

The complete system simulation is shown in Figure 16. Features such as detected corners, bounding box coordinates of the marker, writing these coordinates in the memory block and finally, matching process which gives the ordered vertices of the detected markers are illustrated in this figure. These vertices are sent to the designed soft core processor in order to find out the pose of the detected markers.

The pose estimation using coplanar PosIt running on the designed processor is $\mathrm{Pose}=\left(\begin{array}{cccc} \begin{array}{c} 1.000265 \end{array} & 0 & 0 & \begin{array}{c} 0.000004 \end{array}\\ 0 & \begin{array}{c} 1.000265 \end{array} & 0 & -\begin{array}{c} 0.000004 \end{array}\\ 0 & 0 & \begin{array}{c} 1.000529 \end{array} & \begin{array}{c} 904.239197 \end{array} \end{array}\right)$, whereas the result of coplanar PosIt without approximation is $\mathrm{Pose}=\left(\begin{array}{cccc} \begin{array}{c} 1 \end{array} & 0 & 0 & \begin{array}{c} 0 \end{array}\\ 0 & \begin{array}{c} 1 \end{array} & 0 & 0\\ 0 & 0 & \begin{array}{c} 1 \end{array} & \begin{array}{c} 904 \end{array} \end{array}\right)$.

**Figure 17:**
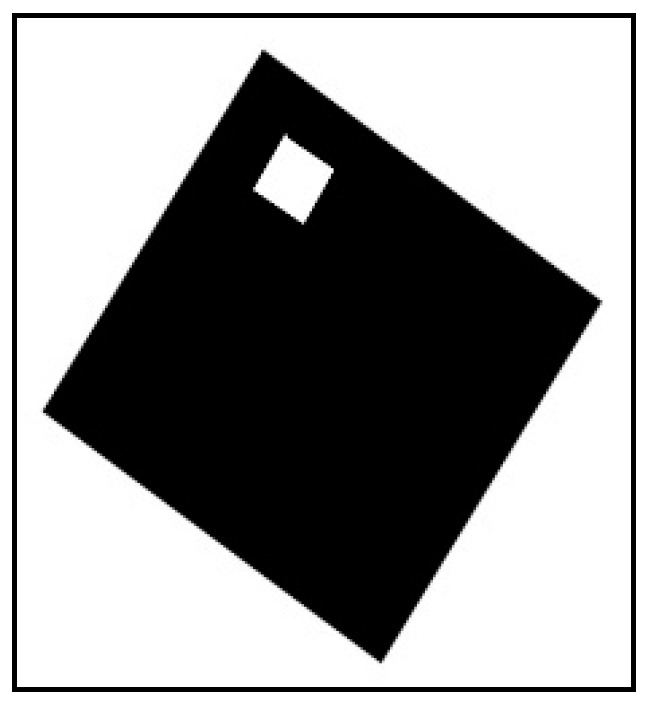
Illustration of an input frame with a rotated marker.

Another example of a rotated marker is shown in Figure 17.

**Figure 18:**
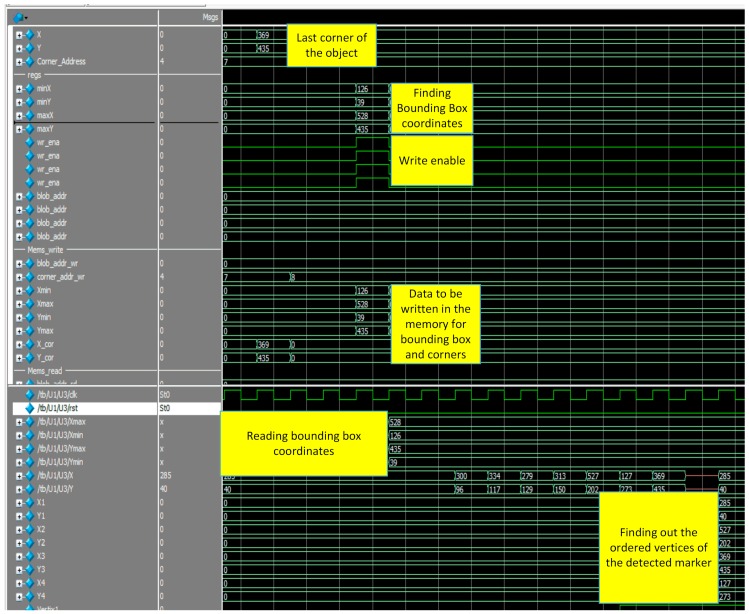
Whole system simulation results.

The whole system simulation for extracting the ordered vertices of the detected marker is shown in Figure 18. The pose estimation using coplanar PosIt running on the designed processor is $\mathrm{Pose}=\left(\begin{array}{cccc} \begin{array}{c} 0.831 \end{array} & 0.555 & 0.051 & \begin{array}{c} 2.896 \end{array}\\ -0.546 & \begin{array}{c} 0.792 \end{array} & 0.277 & -2.931\\ 0.113 & -0.258 & \begin{array}{c} 0.961 \end{array} & \begin{array}{c} 232.437 \end{array} \end{array}\right)$ whereas the result of coplanar PosIt without approximation is given by $\mathrm{Pose}=\left(\begin{array}{cccc} \begin{array}{c} 0.831 \end{array} & 0.555 & 0.052 & 2.896\\ -0.545 & 0.791 & 0.278 & -2.921\\ 0.113 & -0.259 & 0.959 & \begin{array}{c} 232.249 \end{array} \end{array}\right)$.

**Figure 19:**
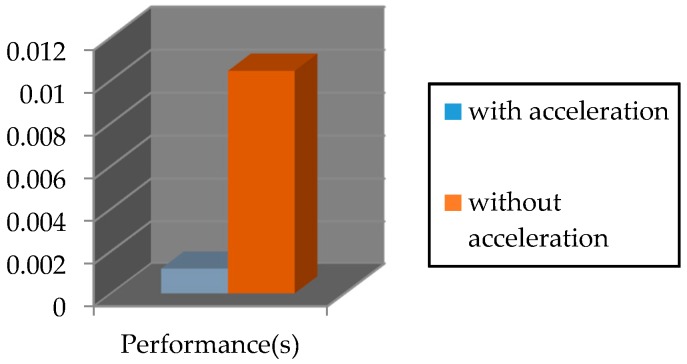
The performance of Coplanar PosIt algorithm.

The required time for executing the PosIt algorithm on Nios II soft-core processor by using FPCI and functions approximation is 0.00118 s. However, the time required for running this algorithm without acceleration is 0.01043 s. This is illustrated in Figure 19.

**Figure 20:**
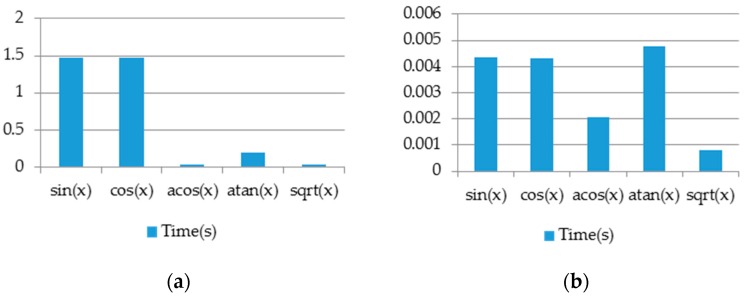
(**a**) Performance of arithmetic functions without using approximation methods; (**b**) Performance of arithmetic functions using approximation methods.

Figure 20 shows a comparison between the trigonometric functions supported by FPCI and the approximated ones. These functions have been tested on an array with 1000 random float-number elements and have run on the designed processor.

**Figure 21:**
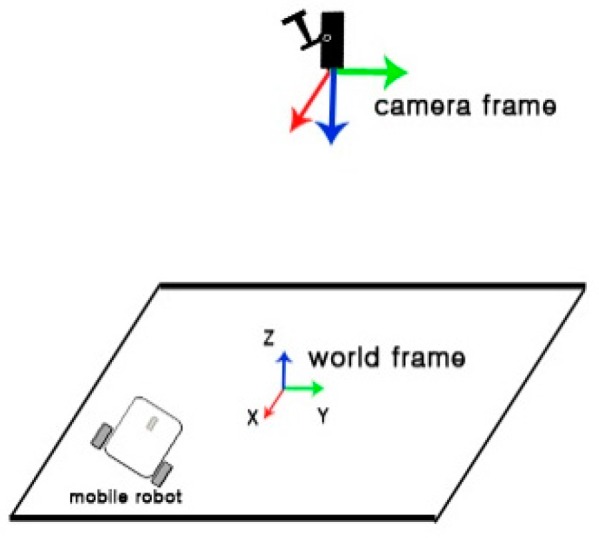
Illustration of camera pose and target pose.

**Figure 22:**
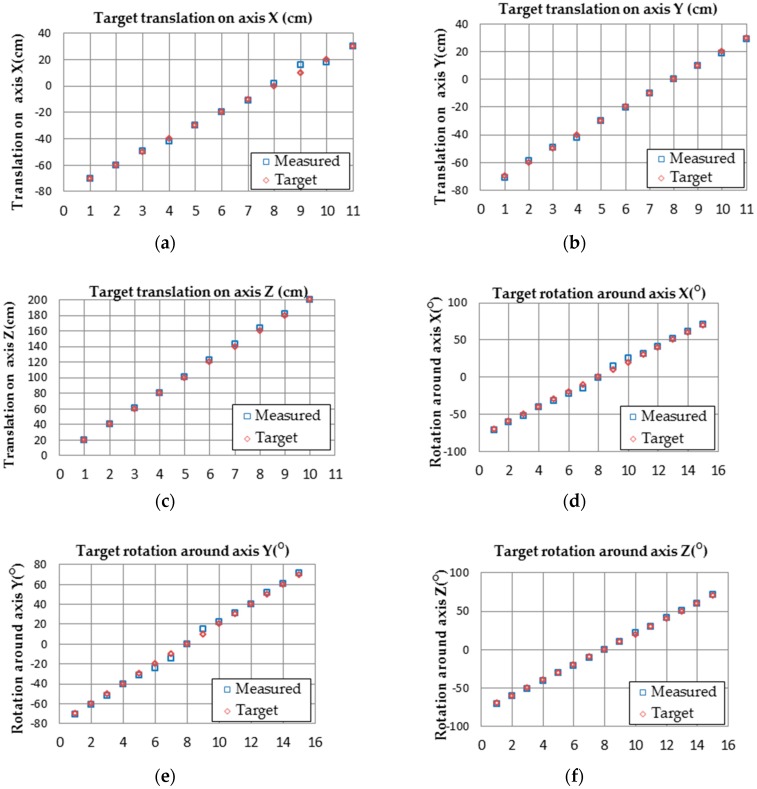
Comparison of pose estimation results. (**a**) Target translation on axis X (cm); (**b**) Target translation on axis Y (cm); (**c**) Target translation on axis Z (cm); (**d**) Target rotation around axis X (°); (**e**) Target rotation around axis Y (°); (**f**) Target rotation around axis Z (°).

In order to test the performance of proposed pose estimation algorithm on Nios II, two scenarios have been considered. In the first scenario, we study the performance of translation and orientation at predefined locations. For instance, the robot moves on the X-axis and all other degrees of freedom are fixed. In other words, the target is either translated or rotated with respect to one axis only. Figure 21 illustrates the pose of the camera and target. Figure 22 illustrates the comparison of estimated pose with the real pose.

The minimum, maximum, and mean error of x, y, and z coordinates and roll-yaw-pitch angles are shown in Table 2.

**Figure 23:**
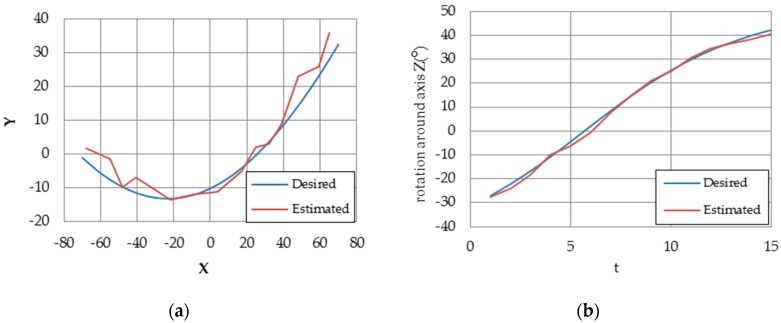
(**a**) Blue line represents the followed path and red represents the estimated pose; (**b**) estimated rotation in red where the real rotation in blue.

In the second scenario, the marker traveled on a predefined trajectory and the estimation error has been recorded and compared with [31]. Figure 23 shows pose estimation and rotation angle results. In [31], the estimation performance was studied with respect to the number of target points (4, 20, 50, 100). However, we estimate the pose using four coplanar target points only. Table 3 shows the comparison of pose estimation error between implemented coplanar Posit algorithm and [31] results. The results shows that our proposed design outperforms the method introduced in [31].

The algorithm mentioned in [34] is used for object tracking, the extracted foreground objects are sent to CPU where simple motion-based tracker is used for object tracking. On the other hand, our proposed algorithm extracts the blobs and corners simultaneously. These information are sent to on-board soft-core processor NIOS II to estimate the pose of the extracted objects using Coplanar-Posit algorithm. Coplanar-Posit algorithm was accelerated by approximating trigonometric functions to achieve real-time results. The performance comparison of the proposed method with reference method is shown in Table 4.

Hardware resources used for building this system are shown in the Table 5. The Cyclone IVE EP4CE115F29C7 has been used for implementing the proposed system.

## 6. Conclusions

In this paper, we have introduced a complete hardware architecture for a real-time visual sensor based on predefined markers. The developed system has two stages; FPGA implementation for image processing algorithms and soft-core processor system using a Nios II core for pose calculation. The proposed system is able to process the input stream of pixels on-the-fly without any need to buffer the frame in the memory. This is because of the running of a FAST algorithm and image segmentation in parallel, use of a simple marker which reduces the matching time, implemention of single pass image segmentation, and the usage of FPCI which accelerates the arithmetic operations on floating point data. Moreover, approximating trigonometric functions played a great role in enhancing system performance. The final system was able to process 162 fps with five markers inside the frame.

## Figures and Tables

**Table 1:** Categories based on the input mask.

P	P1	P2	P3	P4	Category
1	0	0	0	0	New blob
1	1	0	0	0	Copy P1
1	1	0	1	1	
1	1	1	0	0	
1	1	1	1	0	
1	1	1	1	1	
1	0	1	0	0	Copy P2
1	0	1	1	0	
1	0	1	1	1	
1	1	0	1	0	Copy P3
1	0	0	1	1	
1	0	0	0	1	Copy P4
1	1	1	0	1	Compare P1 and P4
1	1	0	0	1	
1	0	1	0	1	Compare P2 and P4
0	0	1	0	0	Finish Blob
0	0	1	0	1	
0	0	0	0	0	NOP
0	0	0	0	1	
0	0	0	1	0	
0	0	0	1	1	
0	0	1	1	0	
0	0	1	1	1	
The remaining cases					Leave Blob

**Table 2:** Relative pose errors of 6-DOF.

Parameters	X (cm)	Y (cm)	Z (cm)	Roll (°)	Yaw (°)	Pitch (°)
Maximum error	5.57	4.5	3.4	1.2	4.7	4.7
Minimum error	1	0	0	0	0.1	0.1
Mean error	2.06	1.95	0.9	0.7	1.8	1.8

**Table 3:** Comparison of relative pose estimation errors.

Method	Translation Error on X-Axis	Translation Error on Y-Axis	Rotation Error
4 target points	3.249	8.7845	2.3101
20 target points	3.3537	8.8294	2.1606
50 target points	3.4167	8.7393	2.1728
100 target points	3.1275	8.9024	2.2154
Coplanar Posit (our implementation using 4 target points only)	2.1924	2.3621	1.0951

**Table 4:** Performance comparison of the implemented systems on FPGA.

Hardware Architecture	Platform	Frame Size	Pixel Depth	Memory Usage	Frequency	FPS
Acevedo-Avila et al. (2016) [34]	Altera Cyclon III	640 × 480	1 bit	307,200	50 MHZ	110
Proposed in this paper	Altera Cyclon IVE	640 × 480	1 bit	307,200	50 MHZ	162

**Table 5:** Hardware resources utilization.

Resources	Used	Percentage
Total logic elements	10,814	9%
Total combinational functions	9.702	8%
Dedicated logic registers	6.683	6%
Total registers	6683	-
Total memory bits	1,703,417	43%
Embedded Multiplier 9-bit elements	11	2%
Total PLLs	1	25%
